# Fostering the Circular Economy with Blockchain Technology: Insights from a Bibliometric Approach

**DOI:** 10.1007/s43615-023-00250-9

**Published:** 2023-01-16

**Authors:** Filippo Corsini, Natalia Marzia Gusmerotti, Marco Frey

**Affiliations:** 1 Istituto Di Management, Sant’Anna School of Advanced Studies, 56127 Pisa, Italy; 2 Department of Management and Law, University of Rome Tor Vergata, Via Columbia 2, 00133 Rome, Italy

**Keywords:** Circular economy, Blockchain, Industry 4.0, Smart city, COVID-19

## Abstract

The circular economy is an emerging paradigm with important economic, environmental, and societal implications. As the world faces such paradigm shifts, new and radical technologies are urgently needed to enable it; blockchain technology can assist to accomplish the aforementioned circular economy shift given its decentralization and distributiveness principles as well as its smart contract capability. This study represents one of the first attempts to analyze those academic research domains together adopting a science mapping technique. By adopting such approach, the study envisages research challenges, highlights important research gaps, and proposes future paths in the blockchain and circular economy fields. Among the others, key findings show that blockchain technology as a tool for promoting the circular economy has been extensively researched at the micro (i.e., firm) and meso levels (i.e., supply chain) more effort on how blockchain can support the development of circular smart cites and measurement tools for providing information to stakeholders and assisting in policy creation expresses the greatest potential in terms of novel research. Moreover, the research suggests that another possible stream of research might be dealing on how blockchain together with physical technologies (e.g., 3D printing, RFID), can support the transition towards the circular economy.

## Introduction

The established linear production and consumption economic model has been recently argued by the circular economy (CE) paradigm [1]. This evolution arises from the need to cope with rapid changes in the global economic system: the scarcity of natural resources, increased attention to the environment, and waste-related difficulties are among the main causes that have led to questioning the current linear model [2]. The concept of CE recalls the “Spaceship Earth” vision coined by Boulding in the mid-1960s [3], who emphasized the limited character of resources. Today, policymakers, managers, practitioners, and academics raise the CE flag as a guiding concept that should inspire legislation [4] as well as business strategies and practices [5]. The CE concept recalls other related aspects such as the cradle-to-cradle approach [6], industrial ecology [7], regenerative design [2], and product life extension [8]. Today’s literature has explored how traditional business models can advance by making gradual improvements to their value chains that promote circular concepts [9]. However, a broader and more consistent shift in the economic system, such as the entire industry becoming circular, cannot rely on gradual approaches. In such a context, there is an urgent need for new and radical technologies to support a larger change [10]. Cleaner manufacturing methods, the use of renewable technologies [11], sharing information and know-how [12], and the creation of relevant regulations and instruments are all goals of the CE that needs to be supported and enabled by technologies [2]. Moreover, there is also a growing demand by customers and governments asking for more transparency from brands [13], manufacturers, producers, and waste management companies throughout the whole supply chain [14]. Finally, the CE calls also for tools and frameworks able to monitor progress in a timely and trustworthy manner in order to lead to better decision-making and real-time feedbacks. Several new technologies (e.g., big data analysis, cloud computing, internet of things) are paving the way to an unprecedented increase in the volume and types of data available, creating several possibilities for informing and transforming society and protecting the environment [15]. The blockchain is one of those technologies. The blockchain technology (BT) has the ability to develop cleaner economic transactional processes and helps achieve the much-needed balance and harmony between the environment, economy, and society due to its make-up as an open-source, peer-to-peer, distributed ledger system with automation capabilities [16, 17]. Although BT was initially created and introduced in 2008 [18], it took several years for various researchers, practitioners, and businesses to see its potential and utility in supporting the transition towards CE. Indeed, in 2015, the main focus on BT was related to the exploration of security and privacy issues connected to this technology and applications strictly connected to payment uses [19]. However, just a few years later, researches and practical applications of BT started to appear in different fields ranging from accounting [20], supply chain management [21], and even connected with medical applications [22]. Nowadays, there are several scientific contributions exploring the use of BT for supporting the CE.

In this context, the aims of this paper are to analyze the use of the BT as the backbone for the development of the CE describing the main areas of research, visualize the most recent trends, and envisage how research in this field can evolve in the near future. Considering that literature in this field is fast emerging, it makes critical to capture the implications of BT development and propose a new stream of research also with bibliometric methods able to deal with a consistent amount of scientific papers. While this method does not provide a comprehensive analysis of all papers of interest, it does provide a systematic and replicable way to map knowledge developments over time and trends of topics of interest in the research literature [23]. Moreover, because it minimizes the researcher’s possible subjectivity, it is a comprehensive technique that gives greater insights than a standard literature review [24]. In this research, using both Web of Science and Scopus research engines, 137 scientific contributes were identified, retrieved, and analyzed. The number of scientific contributions analyzed is six times higher than the contributions identified and analyzed by Böckel et al. [16] utilizing a systematic literature review in assessing the same field of analysis.

The paper is organized as follows: the next section provides an overview of the blockchain and the CE paradigm. The third section presents how data were collected and treated. Then the fourth section presents the results and pathways for further research. Finally, in the conclusions, the study’s most important contributions are emphasized.

## Literature Review

### Defining the Blockchain

Blockchain is a technology allowing the creation and management of a large distributed database for arranging transactions between multiple nodes on a network [25]. The blockchain is structured in blocks that are linked to each other in a way such any transaction initiated on the network must be validated by the network itself [25]. This means that each node is called to control and approve all transactions by creating a network that allows complete traceability of those transactions. At the same time, each block is also an archive for all transactions approved by the network considering the fact that those blocks are unmodifiable and thus immutable [26].

In general, many types of blockchains exist based on the data that is handled, the availability of that data, and the activities that can be performed by the user. The public, consortium, and private are examples of this.

According to Zheng et al. [27], a public blockchain contains all data that is available and transparent to the whole public. Some sections of the blockchain, however, might be encrypted to protect a participant’s anonymity. Bitcoin and Ethereum are two examples of blockchains.

On the other hand, only a limited number of users may participate in the distributed consensus process in a consortium blockchain [27]. Those users might be employed in a single industry or across multiple industries. When a consortium blockchain is developed inside a single industry, it is somewhat centralized and available to the public. On the other side, a consortium of industries (for example, governmental organizations) is made available to the public while yet maintaining a somewhat centralized trust.

Finally, only selected nodes can join a private blockchain network. As a result, it is a decentralized yet centralized network [27, 28]. Private blockchains are permissioned networks that limit which nodes are allowed to make transactions, execute smart contracts, or function as miners. They are overseen by a single entity, which is a trusted third party. It is only used for personal reasons.

The blockchain appeared together with the idea behind a decentralized network to process and verify monetary transactions named Bitcoin [18]. The Bitcoin makes use of the BT to enable payment transactions between strangers without the need for a third-party financial intermediary. In its first idea, then the blockchain had just some potential applications mainly related to cash in detail as digital payment systems, currency transfer, and remittance payments.

In the last years, the use of the BT moved away from its original application (i.e., payments) to embrace wider applications [29–31]. Smart contracts are one of those. Smart contracts are transaction protocols that facilitate the negotiation phases or the performance of a contract. The idea behind this protocol dates back to the 90’ when Szabo [32] presents for the first time the potential benefits (e.g., minimizing the need for trusted intermediaries, minimizing the occurrence of accidental exceptions, minimizing transaction costs) of this application in important contracting areas such as rights management, payment systems, and credit. According to Rozario and Vasarhelyi [33], when data are received and evaluated in terms of the pre-defined contractual terms, a smart contract is triggered. If the pre-set conditions are met, the needed outcome is generated,otherwise, an error message is sent to all nodes on the smart contract blockchain. The preliminary application of smart contracts in business started to appear in 2010. For example, Narendra et al. [34] explore the implementation of smart contracts in business establishing a collaboration network with peers (i.e., virtual enterprises) to overcome the potential conflicts that might arise during collaboration. Other examples are related to the energy field,indeed, the idea of managing electricity transactions based on smart contracts and blockchain is recurring in recent research [35]. More recently, the introduction of BT has provided a distributed system—secure and interconnected—to facilitate the implementation of 3D manufacturing [36], IoT technologies [37], additive manufacturing [38], and other technologies [39, 40].

### The Circular Economy, Barriers, and Enablers

Defining CE in not easy; indeed, there is currently a lack of agreement on this subject. As Kirchherr et al. [41] showed that a substantial percentage of the publications available today articulate a number of principles that build the groundwork for the transition to the CE. The 3Rs principles (reduce, reuse, and recycle) are the most prevalent and commonly referenced set of principles [2, 42, 43]. In some circumstances, recovering was included as a fourth principle. Reducing includes early creation and consumption process actions including redesigning, rejecting, and reevaluating material utilization. Reusing includes both product repair and operations to close material loops. Recovering involves energy recovery when materials are burnt, while recycling includes repurposing garbage and other resources [41].

The CE has been also defined as a cycle of resource extraction, transformation, distribution, use, and recovery of materials [44, 45]. To begin, businesses extract resources from the environment and convert them into products and services,those are then distributed to customers at retail locations or to other businesses, and customers use them. Finally, CE suggests that the loop should be closed by recovering those products enhancing the reuse of materials rather than discarding or disposing them [44].

However, many authors underline also other relevant aspects that define CE. For instance, Howard et al. [46] and Morseletto [47] stress about the importance of regeneration in the CE definition. Regeneration is the process to rethink resource generation, consumption, and disposal activities in such a manner that the wider ecosystem’s health is maintained and biological nutrients are restored to the biosphere. Regeneration can take several forms, including the valorization of waste or the generation of energy using refuse-derived fuel [48]. Transitioning to renewable energy sources is also a viable option for regeneration [49].

Other authors identified CE with a closed-loop system [4, 50]. Recycling, remanufacturing, and biochemical extraction from organic waste are all examples of loops, in which outputs become inputs in the economy. Among the actions that can be undertaken for closing loops, industrial symbiosis can be seen as one of the most promising [51]. Industrial symbiosis consists of transforming the waste of different processes and industries into other industries, enabling the transition to closed-loop systems [52, 53]. According to Chertow [54], IS implies inter-firm collaborations for exchanging and reprocessing of wastes and other excess resources from one firm into valuable inputs for another. Formally, it is the physical exchange of materials, energy, water, and by-products among geographically proximate firms [54], p. 314).

When we expand the research of a definition to the grey literature, it gets even more diverse. The Ellen MacArthur Foundation, a non-profit organization committed to the comprehensive development and implementation of the CE, has proposed three circular value creation principles to define CE [55]. Firstly, by dematerializing and virtualizing utility distribution as well as adopting renewable technologies and processes, the finite supply of natural resources can be preserved. The second principle suggests product and process optimization through recycling, refurbishing, and remanufacturing to recirculate resources without losing their value. Finally, the third principle suggests the utilization of renewable and resilient resources [55]. Ellen MacArthur Foundation proposed also the ReSOLVE framework to define CE [55]. The ReSOLVE framework tries to categorize CE practices in a comprehensive way [56], and it has been used by many researchers in recent years to better comprehend and analyze CE practices [49, 57, 58]. The ReSOLVE framework includes six different operational actions for implementing CE: regeneration, sharing, optimization, loop, virtualization, and exchange.

In addition to definitory aspects, literature on CE dedicated much attention to drivers towards the CE and barriers impeding a full transition towards this paradigm. For instance, De Jesus and Mendonça [59] identified factors, such as a lack of technology solutions and financial obstacles like high investment prices and linear lock-ins, as the biggest obstacles to the shift towards the CE. On the other hand, variables such as governmental policies, environmental concerns of consumers, and demand for environmentally friendly goods are what propel the change [59]. In such a context, also Prieto-Sandoval et al. [60] illustrated how eco-innovation determinants (regulatory and policy, supply-side activities, and demand-side needs) connect to the CE idea in order to emphasize the significance of innovation for a transition to the CE. Their analysis revealed that while customers (the demand side) are deemed vital for embracing eco-innovation and accelerating the transition through their altered behavior, law and policy provide the legal underpinning to increase circular supply [124]. Several other publications stress the necessity of innovation and a technology-oriented approach to correctly implement those actions [2, 10]. According to Bouton et al. [61], CE enabled by modern technologies may help Europe boost its resource productivity by up to 3% throughout the economy. In such a framework, BT can be considered a social technology or a tool for collaboration in the context of the CE [62]. It aids in the coordination and connection of various distributed databases, all of which may be updated at the same time and are available to all stakeholders.

In regard to the preceding description of CE, BT can efficiently cater to the aspects of sharing, optimization, and virtualization. As a result, a wide range of regenerative digital alternatives for the CE’s objective of sustainability become available. For instance, by using a decentralized approach of value production and circulation rather than the traditional value creation approach, the CE can profit from simultaneous collaboration [62]. Through its principles of decentralization and distributiveness [63], as well as its functionality for smart contracts [64], BT can help achieve several of the above-mentioned CE targets.

At present time, only Böckel et al. [16] conducted a literature review of BT and CE of academic researches and other documents. Their research which highlights patterns of interests and opportunities for research and practice as well as mutual blind spots that need to be addressed in either domain by conducting literature was of 57 documents (of which 20 academic researches). Major findings underlined by the authors suggest a lack of a clear terminology for BT types and that a thorough examination of the potential benefits and challenges of BT for the CE and its links to sustainable development is critical.

## Methods

The methodology adopted in order to perform the analysis is a bibliometric mapping approach. The main objective of this approach is to visually represent relationships between items of interest. In detail, such methodology has been used in several fields [65–67] in order to explore the relationships and evolution in academic research over the time and produce either:

- a visual representation of the state of the art in a research area;
- an orientation for scientists and practitioners in expanding their area of interest on the topic;
- a comprehensive analysis of future approaches in developing a field of research.

Although a few recent bibliometric researches have addressed the CE research field [68], et al., 2018) as well as the BT field [19, 69], so far, no studies have analyzed those research domains together. In various ways, this bibliometric analysis is unique. To avoid subjectivity and prejudice, it used a science mapping technique (consisting of bibliometric literature) in the growing research topic of BT and CE. Second, this research goes beyond the scientific mapping technique by delving further into the highlighted research themes. Third, it examines research issues, identified significant research gaps, and suggested future directions in the BT and CE domains.

### Data collection

**Fig. 1:**
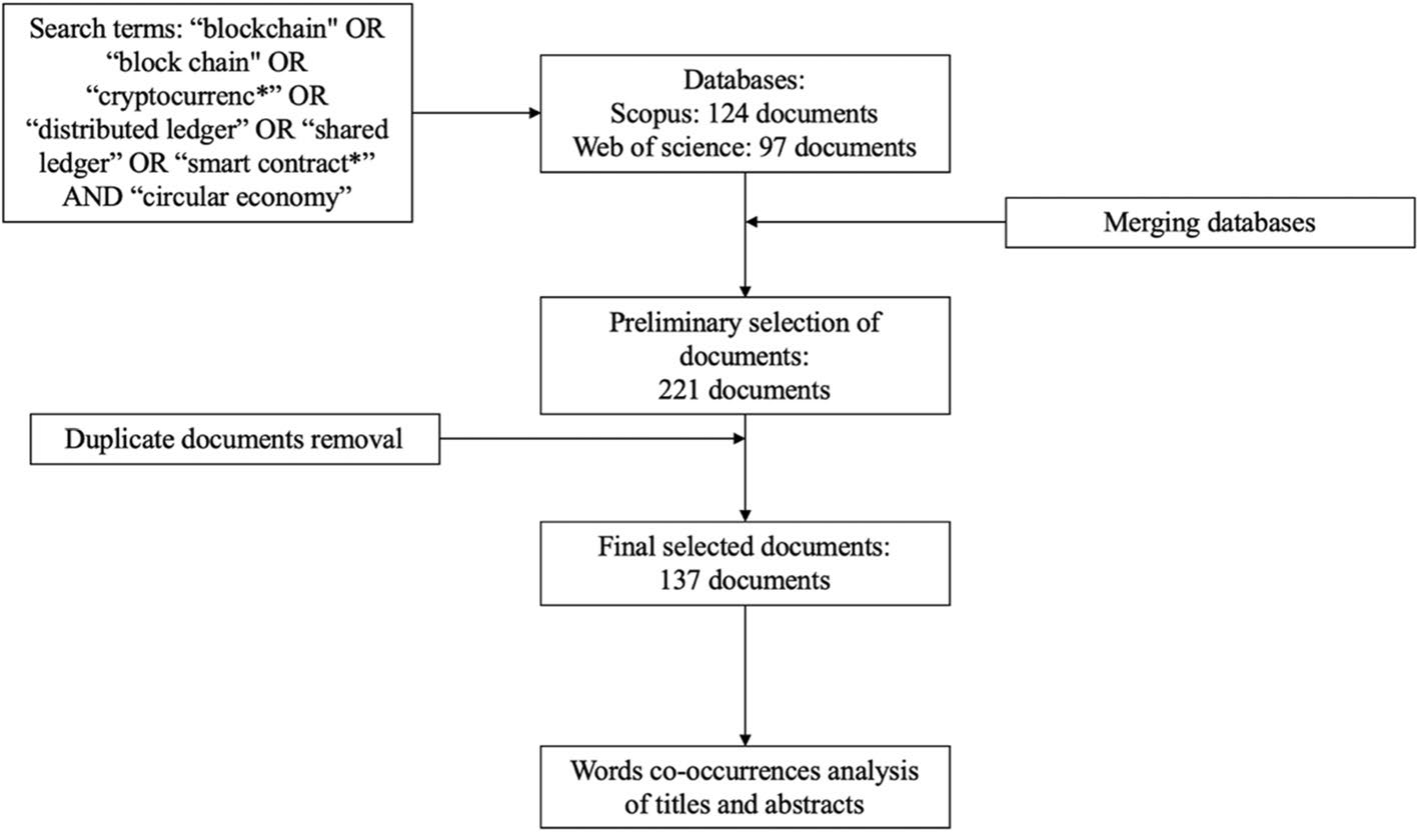
Outline of the research design

In order to address the study’s research aims, the first step was to identify the appropriate papers dealing with BT and CE. In more detail, the research design depicted in Fig. 1 has been followed.

Data were gathered from the two main scientific citation and indexing services, namely, Web of Science (WoS) and Scopus. In comparison to EBSCO, Google Scholar, or others, Scopus and WoS databases ensure a greater range of high-quality, peer-reviewed journals on the subject under investigation [70].

Because this analysis aims at understanding a relatively recent phenomenon, no precise time frame has been selected. For the same reason, it was decided to not limit the search just to articles published in academic journals, but also those presented in conferences and available into books that were indexed on the two citation and indexing services. Only English-language publications were considered.

Publications were selected through a keyword filtering criterion as both WoS and Scopus allow for keyword search. In more detail, to retrieve all relevant papers, the Scopus and WoS keyword searches were adjusted to include titles, abstracts, and keywords.

Firstly, a shortlist of keywords has been selected after having drafted the first outline of publications on the topic [2, 16, 19, 25, 45, 71]. The keywords selected at this stage were used to perform the papers selection using the following string:

“blockchain” OR “block chain” OR “cryptocurrenc*” OR “distributed ledger” OR “shared ledger” OR “smart contract*” AND “circular economy”

The initial search generated 221 publications (124 from Scopus and 97 from WoS), but when the results were refined by removing duplicates, the Scopus and WoS databases yielded 137 papers.

Table 1 provides some descriptive information about the publications collected through the research process in terms of publication year and document type. Data contained in Table 1 suggest the novelty of the research field and the relevance of sources different from research articles published in peer-reviewed journals (51.82%).

**Table 1:** Descriptive statistics for the retrieved publications

Publication year	Number of documents	Percentage	Document type	Number of documents	Percentage
2022	33	24.09%	Articles	66	48.18%
2021	59	43.07%	Conference papers	46	33.58%
2020	27	19.71%	Reviews	12	8.76%
2019	11	8.03%	Book chapters	11	8.03%
2018	7	5.11%	Others	2	1.46%
Total	137	100%	Total	137	100%

### Term Maps of the Research Domain

The chosen methodological approach aims at identifying the structure of all the available contributions related to the field of BT. In particular, by producing a visual representation of this research field, the paper aims at providing an overview of the whole research area, the different subtopics, how those subtopics are linked together, and especially a temporal trend of those researches. The fundamental assumption is that word co-occurrences reveal the underlying intellectual structures of research dynamics and consequently can represent a useful technique for assessing real word applications. This analysis represents a useful means to investigate the orientation of future research lines and business application within this field. In detail, VOSviewer [72] has been used to create and visualize the bibliometric landscapes related to this research area. In order to refine the results, terms that were too general (e.g., “research,” “methodology,” “outcome,”) have been iteratively excluded. The text-mining routine refers to the method developed by Van Eck and Waltman [73]. Accordingly, to create a term map, VOSviewer goes through the following four steps: step 1 [74], identification of the noun phrases by performing part-of-speech tagging through the Apache OpenNLP toolkit; then, converting plural noun phrases into singular ones by means of a linguistic filter; and step 2, selection of the most relevant noun phrases through the assessment of the Kullback-Leiber distance [75]. Between the distribution of second-order co-occurrences over all noun phrases and the overall distribution of co-occurrences over noun phrases,the higher the distance between the two distributions, the higher the relevance of a noun phrase; and these highly relevant noun phrases are clustered together; step three, mapping and clustering of the terms, by using the unified framework for mapping and clustering [76], step four, visualization of the mapping and clustering results. The resulting maps illustrate the relations between relevant terms. It must be noted that words in larger or smaller fonts represent respectively those occurring most frequently across the identified publications or those occurring less frequently; distances between words distributed on the map are such that the smaller the distance between two terms, the higher their co-occurrence; linkages between words show the connections between words mostly occurring together in the selected documents; finally, colors represent the temporal dimension in publications.

Finally, a term map representing the whole research field composed by the 137 papers collected has been produced.

### Overlay Representation of Journals in a Global Map of Science

In order to examine the interrelations in the field of BT and CE, we used an overlay framework that allows to represent the set of retrieved papers on a global map of science based on 20.554 academic journals [77]. This process allows for producing a journal map where the journal names of the 66 papers published in academic journals are highlighted with colors in the map, and the size of each journal as a node is represented proportionally to the number of occurrences.

The Rao-Stirling diversity index was developed as a measure of interdisciplinarity in the BT and CE research field [78, 79]. The Rao-Stirling index considers variety, balance, and distance between categories giving greater weights to pairs of references in more distant categories. The Rao-Stirling index has the characteristic to integrate close and remote interdisciplinarity by weighting them in one formula.

## Results and Discussion

**Fig. 2:**
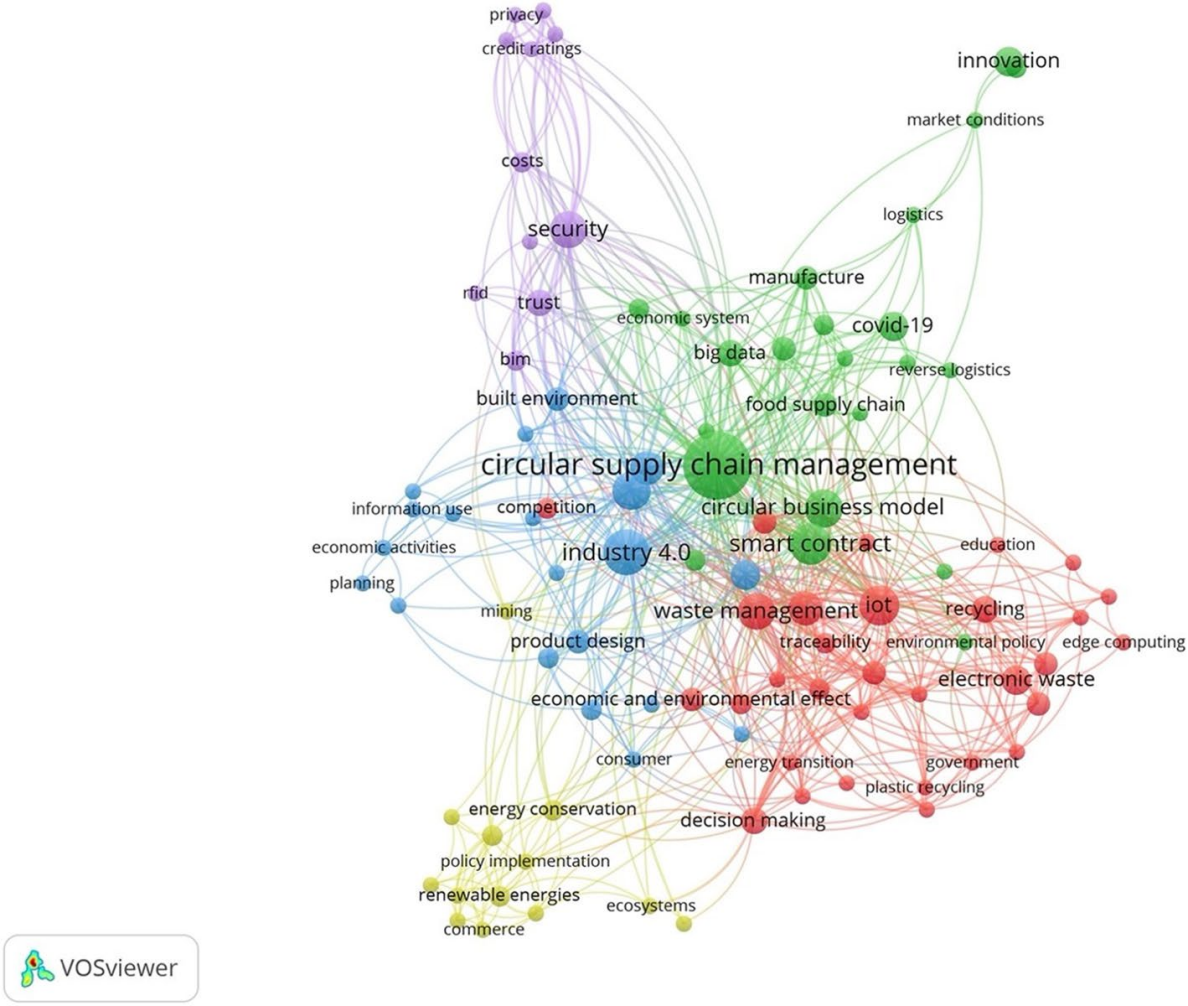
Term map of the research domain

The map shown in Fig. 2 identifies five major areas of research.

The green cluster groups business-related keywords and researches conducted with the aim of exploring the use of BT to organize the supply chain according to CE principles but also how BT can be a useful tool to revise the business model to adopt a more circular one. Studies exploring the use BTs in circular supply chains aim mostly at introducing novel models based on BT aiming at solving traditional supply chain problem such as the relationships between supply chain members or the absence of information for consumers regarding product origins.

For instance, Zhang [14], adopting a multiple-case approach, analyzed the role of BT in the steel supply chain industry as an instrument to foster the CE by encouraging inter-organizational trust, data transparency, and interoperability. The authors stress the fact that BT contributes to a resilient steel supply chain in reducing risks and uncertainties with its transparency and immutability function. Other relevant case studies, for instance, investigate such issues in connection with the textiles and clothing supply chain [80] and the food supply chain [81].

Nowadays, the COVID-19 pandemic has brought to light the flaws in supply chains as well as the limitations of present technologies. Interestingly to notice, some of the researches related to keywords belonging to this cluster explored also how businesses create new skills and abilities by grounding on CE principles and BT in responses to supply chain disruptions during COVID-19 [17, 82, 83]. For example, Nandi et al., [84], using the resource-based and resource-dependence theoretical approaches of the firm, explored firm responses to supply chain disruptions during COVID-19. In more detail, the authors investigate how firms develop localization, agility, and digitization capabilities by applying their critical CE and BT-related resources and capabilities that they either already possess or acquire from external agents. Their findings show significant patterns on adoption levels of the BT-enabled CE system and localization, agility, and digitization capability development [84].

Finally, the cluster suggests the application of the BT in supporting reverse logistics operations for returned, damaged products and products reaching the end of life as an enabling technology to implement take-back programs by firms (i.e., initiative arranged by a firm to collect its used products from consumers and reintroduce them to the original processing and manufacturing cycle) [85].

According to Narayan and Tidström [86], one of the qualities that make BT so extensively applicable is that it allows for intra-organizational collaboration to be facilitated. In more detail, by implementing this technology, businesses can tackle the problem of managing information flows inside a corporation and assuring their reliability. Researches in this field then explore also how the technology can support new business models aiming at revising the traditional production and consumption cycles relining heavily on product and service information [87]. BT-based business models can support managers into rethinking their orthodoxies about value creation and value capture [88] in general, with a particular emphasis on the relationships among customers, offer, infrastructure, and financial viability. Then, the new paradigm shift comes true in moving from a static to a dynamics business model, where products continuously monitor and update themselves also with the help of other technologies. In summary, researches in the green cluster show that the BT offers powerful options for embracing the CE business model to those firms willing to undertake this radical shift. To do so, firms will find support in the BT as a means to redesign their value chains, embedding new ways to move from the customer’s use, and return of the end-of-life product at very low transaction costs. This finding case is in contrast with Böckel et al. [16] supporting that the study for new and circular business models through BT is also considered in academic research and not only in practitioners’ documentations.

The red cluster at the bottom left of the map is connected with issues related to the end-of-life management of products (i.e., waste management activities). Topics on this cluster are strongly connected with the Internet of things technology (IoT). The IoT is a fast-growing industrial field aiming at transforming business activities, cities, and homes making them smarter by embedding them with electronics, software, sensors, and connectivity that enable a wide variety of objects to collect and exchange data. According to Biswas and Muthukkumarasamy [89], embedding the BT in the IoT ecosystem framework will enable a more flexible networking paradigm where different devices could rely on a BT infrastructure to identify and authenticate devices for collecting and sharing data. Waste management research seems strongly focusing on BT and IoT to improve performance especially regarding the plastic waste and waste electrical and electronic (WEEE) management.

One of the most important bottlenecks for the uptake of CE strategies is a lack of trustworthy information on the availability, amount, quality, and compatibility of recycled feedstocks. Manufacturers are not incentivized to purchase recycled feedstock without such trustworthy information [90].

In such a framework, researches addressed some of the structural challenges emerging due to the lack of technologies in plastic waste collection and recycling processes and the lack of reliable data about recycled plastics [91]. In particular, researches focused on how information regarding the appropriateness, quality compliance, bid-and-offer price, and availability of recycled plastic feedstock can be acquired by IoT and securely communicated between collectors, recyclers, and manufacturers utilizing a consortium-based BT. Another issue tackled by researchers is connected with WEEE management. WEEE prevention is particularly important in the context of CE since it is a rapidly rising waste stream that contains both environmentally harmful compounds and valuable and rare materials. Also, in this case, the adoption of a properly combined IoT and BT can assist producers in maintaining product control until EEE end-of-life, while also boosting CE strategies and assisting decision-making management options (e.g., recycling vs energy recovery) [92]. For instance, Magrini et al. [93] investigated how the use of a proper combination of IoT and BT can help the producers monitor EEE all along their life cycle and decide the appropriate action to undertake such as reuse, repair, remanufacturing, or recycling. Interestingly, other waste streams, such as, for instance, toxic waste, textiles, construction, and demolition waste, seem not to be adequately investigated by current research, even if BT can offer relevant benefits also in those sectors.

The yellow cluster groups issue is strictly connected with energy generation and management in the context of CE. The transition towards CE call for the adoption of natural energy resources and for reducing energy consumption. Energy distribution networks have become increasingly sophisticated as the trend toward the utilization of renewable energy resources has grown. As a result, efficient energy distribution, unlawful energy consumption, and individual energy producers’ entry into the market are some of the most pressing concerns nowadays. In this context, BT and its integration with renewable energy systems are discussed widely in the literature [49, 94, 95]. For instance, Zhu et al. [95] suggest that by providing a decentralized trading mechanism, BT can facilitate sustainable energy consumption and achieve a CE. In detail, their research analyzes how China can employ BT to reform its energy sector discussing the advantages and disadvantages of applying of such a solution. The authors suggest that transparency of energy usage may be promoted by using the BT and a smart contract. Other researchers, e.g., [49], suggest that the adoption of BT can reduce the needs for active intermediaries in the market once BT is implemented, dramatically lowering transaction costs. Flexibility, openness, and decentralization are all characteristics of BT that can foster individual renewable energy producers’ entry into the market [94]. In more detail, BT enables parties to connect with one another in order to carry out automated energy transactions such as auctions, bidding, and payment,each node in the BT negotiates a spot price, transmission channel, and transaction priority allocation. Finally, in the context of CE, researches exploring the energy-related issue in connection with blockchain applications are also investigating consumption issues connected with such a technology [96]. For instance, Manganelli et al. [97] suggest that widespread adoption of those blockchain applications might have significant environmental repercussions, thus hindering the CE transition. Even if energy generation and management represent another consistent area of researches, topics belonging to such cluster seem quite independent and less interlink with other fields of research in CE.

The blue cluster groups issue is mostly connected with exchanging of information of products all along their life cycle, from the design phase through the usage of consumers including the condition of the product after the first use and or reuse potential. The cluster is thus strictly shaped by the topic of Industry 4.0 a paradigm that was first introduced in Germany in 2011 at the Hannover Messe [98]. Industry 4.0’s fundamental goal is to attain more accuracy and precision, as well as a higher level of automation [99]. Industry 4.0 encompasses a vast variety of notions that are impossible to categorize or distinguish precisely [100]. The major goal of Industry 4.0 is the facilitation of real-time interconnection between machines, digital devices, transactions, and different stakeholders such as purchasers and customers with digital technologies (e.g., big data, additive manufacturing, simulation, IoT, etc.) [101, 102]. In such context, Industry 4.0 also has forward-thinking consequences in allowing the adoption of CE as suggested by many researches [11, 83, 102–105]. Industry 4.0 and CE would allow more flexible, sustainable, self-organized, secure, interoperable, and heavily reliant information flow based on communication technology [106]. BT can be an additional technology for supporting Industry 4.0. Current researches exploring the application of BT for the CE often investigates multiple digital technologies within the same research, namely, artificial intelligence; big data, digital twin, edge computing, IoT, and machine learning. In more detail, those terms were found in 48 publications retrieved (35.03%). Some of those publications suggest that great benefit can be achieved in adopting BT together with other technologies. For instance, Alves et al. [80] examined current approaches to traceability in the textile and clothing value chain suggesting how to exploit BT, IoT, and digital twins for improving the CE performances. Another relevant example is represented by the research conducted by Ada et al. [107], in which the authors assessed the food supply chain by proposing Industry 4.0 solutions to forester circularity. In particular, the authors underlined how BT and big data analytics can provide the necessary support to establish legal systems and improve environmental regulations since transparency is a crucial issue for taxation and incentive systems. More details and examples are presented in Table 2.

**Table 2:** Connections between BT and other digital technologies for CE

Digital technologies	Technology description	Examples of researches exploring CE, BT, and other digital technologies
Artificial intelligence	Artificial intelligence (AI) is a broad field of computer science that focuses on creating intelligent computers that can accomplish activities that would normally need human intelligence	Chauhan et al., [108]
Big data	Big data refers to data collections that are too massive or complicated for typical data-processing application software to handle	Ada et al., [107], Çetin et al., [109]
Digital twin	A digital twin is a virtual representation of a physical item that acts as its real-time digital equivalent	Alves et al., [80], Teisserenc & Sepasgozar [110]
Edge computing	Edge computing is a distributed computing paradigm in which processing and data storage are brought closer to the data sources	Damianou et al., [111], Andersen & Jæger [112]
IoT	IoT refers to physical objects that are equipped with sensors, computing power, software, and other technologies to connect and exchange data with other devices and systems through communications networks	Hatzivasilis et al., [113], Lawrenz & Leiding [114]
Machine learning	Machine learning represents the study of computer algorithms that can learn and develop on their own with experience and data	Hoosain et al., [115], Conforto [116]

Finally, the purple cluster groups keywords dealing with problems and drawbacks in using the BT for supporting the CE. While BT has huge potential to help in the transition to the CE, the term map shows that the technology faces a number of technical problems also connected to CE. Security, such as cyber-attacks and issues related to BT use in CE contexts, were highly debated in research [85], as well user privacy is also quite debated in literature connected with CE [117]. The trustworthiness of actors in networks seems to be another relevant aspect investigated by researchers. For instance, Wu et al. [118] built a buyer’s and seller’s credit ratings, allowing the two parties to use credit ratings to choose a reliable business partner in the context of CE.

Other technical challenges include high development costs and limited scalability; all of those are also largely mentioned in researches [119]. Moreover, the lack of technical understanding of BT by users and companies in order to use it for the CE was an addressed obstacle in the researches under investigation [21].

### Future Research Pathways

Several researchers find an agreement that CE is divided into three stages of study and implementation: micro, meso, and macro [120]. Companies are focusing on their own CE adoption at the micro level. The meso level contains businesses that are part of a supply chain adopting the CE paradigm. Finally, through the development of environmental regulations and institutional influence, the macro level is heavily focused on the creation of circular cities or regions. Yet, the CE represents a systemic change, implying a network-wide adjustment [121]. From the analysis conducted, the adoption of BT in support of the CE at the micro and meso level seems to be quite investigated by researchers. On the contrary, how BT can support the adoption of CE at the macro level is a research area that is still largely uncovered. Considering the rising interest in the concept of the smart city, as a form of “instrumented, interconnected and intelligent city” [122] and the wider adoption of BT in the smart city context [123], future research can deepen this exploration. In more detail, future research can explore how BT can foster the adoption of the CE principle in a smart city context intensifying researches on the novel concept of “smart circular city” [124].

Some recent research in the field of CE suggest that it is vital to build a measuring mechanism in order to determine if CE adoption is contributing to meaningful change [125, 126]. Indicators and other measurement approaches have the potential to condense the tremendous complexity into a manageable quantity of valuable information. For instance, at a macro level, CE measures are becoming more widely acknowledged as a beneficial instrument for providing information to stakeholders and assisting in policy creation. In such a context, academic research can deeper explore how BT can be exploited to CE measuring purposes.

Another possible stream of research arises from the fact that analysis of how BT together with physical technologies can support the transition towards the CE is also under-dimensioned at present time. According to Lacy et al. [127], physical technologies are, for instance, 3D printing, robotics, physical markers (e.g., QR code), and virtual reality. While few researches on the usage of physical markers such as radio-frequency identification (Rfid) are emerging in the literature [128, 129], other physical technologies are almost unexplored. For instance, BT can ensure that the blueprints for 3D printed items are made of certain recycled material and are comprehensive and compatible, as well as securing a manufacturing model for industrial intellectual property owners and producers.

Considering that from the analysis emerge that BT for supporting the CE is not immune from problems and drawbacks (as presented in the analysis), future research can focus on addressing those existing issues and new ones. For instance, one recent issue acknowledged by Caldarelli [130] is the so-called oracle problem. Oracles are the means of communication between blockchain and the physical world, and unlike blockchain nodes, they are centralized and must be trusted [130]. Because there is unlikely to be a perfect solution to this problem in the near future, the danger of incomplete and erroneous data inputs on the blockchain for CE applications is real. Thus, future researches can investigate how to securely guarantee the physical connection with the BT for instance with certifications tools for oracles.

Moreover, several keywords in the term map suggest that the benefits of BT for the CE were largely explored in academic research. In particular, a focus on the business and environmental advantages is assessed in researches dealing with the supply chain and business models. This field of research seems thus evolving similarly to most of CE-related researches that have primarily focused on the environmental and economic dimensions, while social aspects such as have only been tangentially and sporadically incorporated into the CE concept [131]. In such a framework, how BT can stimulate also social benefits (i.e., better labor practices, respect of human rights, and community well-being) in the context of CE are under-evaluated in academic literature. Future researches thus can also focus on such an aspect or even better explore how BT can allow considering the trade-off between environmental and/or economic gains and the loss of certain social advantages.

**Fig. 3:**
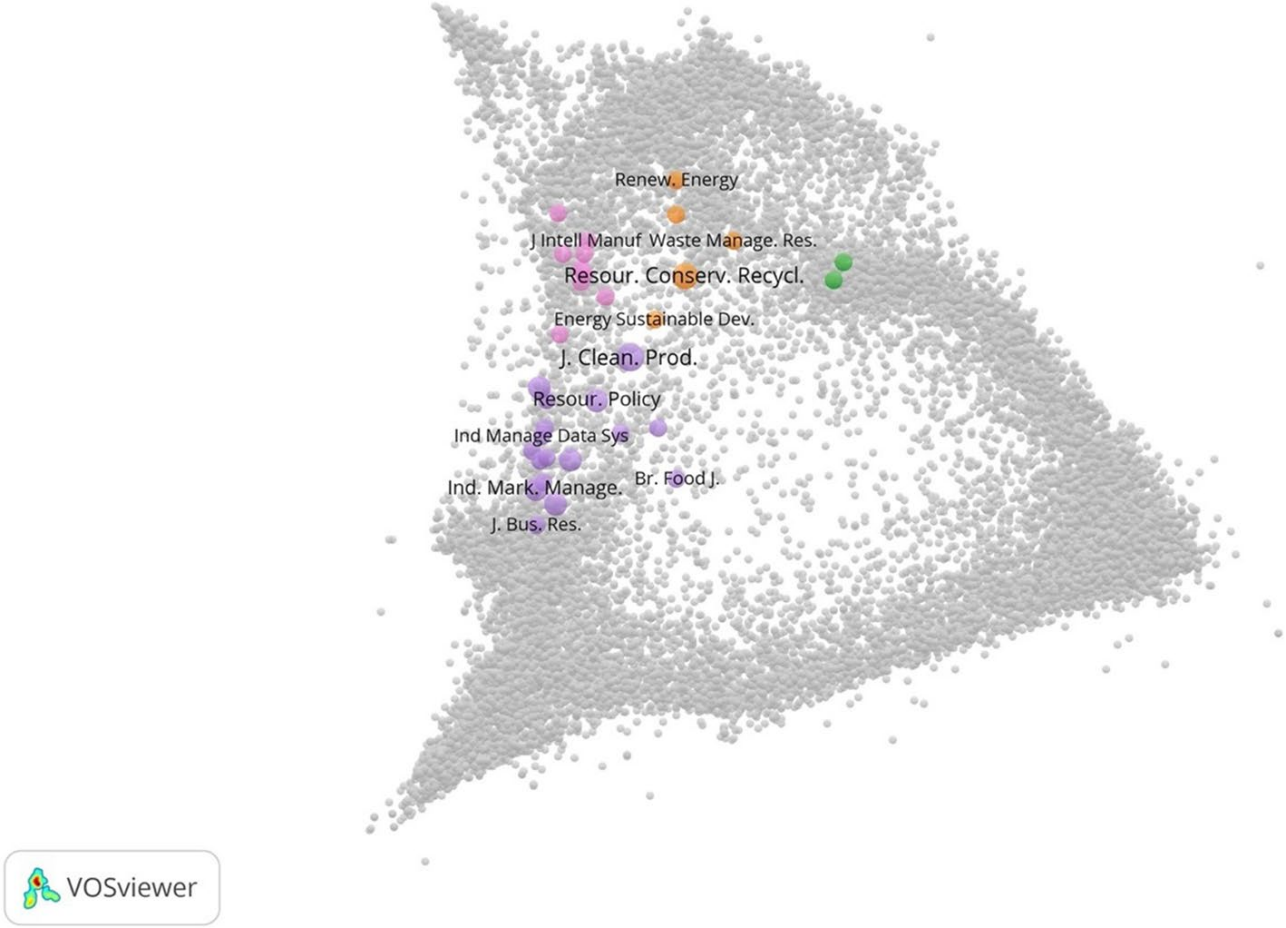
Term map of the research domain

Finally, in order to deeper investigate the research field and formulate future research avenues, an overlay representation of journals in a global map of science has been produced. Even if the papers presented in this map are only 66 (i.e., those published in academic journals), this approach can be useful to deeper assess the research domain at this initial stage. As presented in Fig. 3, contributions to the research domain are mostly published in engineering-related journals, business-related journals, and in some transdisciplinary journals covering environmental and sustainability research. The low level of interdisciplinarity can be also assessed by the Rao-Stirling diversity index which is equal to 0.11.

The transition to the CE is also supported by material science, chemical, and biological science [132, 133], and BT can help in testing, engineering, or even connecting materials,in this context, more interdisciplinary research touching also these disciplines is thus needed.

## Conclusions

BT and the CE are two new paradigms with significant consequences for business and society. This research represents one of the first attempts to analyze those research domains together by adopting a science mapping technique. Moreover, the research examined research issues, identified significant research gaps, and suggested future directions in the BT and CE domains.

The clusters identified in the term map suggest that BT as a tool for supporting CE has been widely explored on a micro (i.e., firm or event product) and meso level (i.e., supply chain). On a macro level, academic researchers are merely dealing with waste management activities that can be supported by BT and other technologies such as IoT. Thus, fostering researches on the potential usage of the BT on a macro level (e.g., city, regions) represents one of the most promising avenues for future researches.

Other researches focused on how BT can support the exchange of product information all along their life cycle also with the help of other typical Industry 4.0 digital technologies. Thus, another possible stream of research is connected with understanding how BT together with physical technologies can support the transition towards the CE (e.g., 3D printing, virtual reality.). Finally, results show also a low level of interdisciplinarity of the research domain at present time, thus would be necessary also to connect more with other disciplines such as material science, chemical, and biological science.

Despite the importance of the results, some possible limitations should be mentioned. Firstly, a limitation could be inherent in the assumptions taken during the keyword selection process; even if the keyword were validated by international experts on the topic, some distortion could be still caused by the data selection process. Secondly, as suggested by Heersmink et al. [134], a map is a simplified representation of the reality and then maps should not be blindly trusted, but rather used as support for experts for the development of insights.

## Data Availability

Data is registered and stored in compliance with the general rules for scientific data management.
